# Biological evaluation and nutraceutical potential of Bambina, a resilient Apulian olive cultivar, through an advanced milling process

**DOI:** 10.3389/fpls.2026.1815003

**Published:** 2026-05-01

**Authors:** M. Volpicella, L. Fiorentino, G.R. Caponio, D. De Angelis, V. Scaglione, G. Tamma, G. Squeo, C. Montemurro, W. Sabetta

**Affiliations:** 1 Department of Biosciences, Biotechnologies and Environment (DBBA), University of Bari “Aldo Moro”, Bari, Italy; 2 National Research Council, Institute of Bioscience and BioResources (IBBR), Bari, Italy; 3 Department of Soil, Plant and Food Science (DISSPA), University of Bari “Aldo Moro”, Bari, Italy; 4 Spin off Sinagri s.r.l., University of Bari “Aldo Moro”, Bari, Italy

**Keywords:** *Olea europaea*, Bambina cultivar, biodiversity, nutraceutical milling, bioactive compounds, antioxidants

## Abstract

**Introduction:**

In Italy, the Apulia region is renowned for its extensive olive biodiversity, which includes a wide range of autochthonous germplasm playing a crucial role in the regional economy and cultural heritage. The preservation and valorisation of this biodiversity are fundamental for sustaining healthy agro-ecosystems, promoting local olive varieties, and enhancing the marketability of high-quality food products. The aim of this work was to characterize and valorise the minor autochthonous olive cultivar Bambina, which exhibits notable rusticity and adaptability to diverse climatic conditions, through a comparative analysis with the well-known and widely appreciated cultivar Coratina.

**Methods:**

A comprehensive characterization of the Bambina olive cultivar was carried out by analysing drupes, leaves, and derived products (pastes and oils). A modified milling extraction process was employed to enhance the retention of bioactive compounds and improve the overall nutritional quality of the oils. Gene expression and enzymatic activity assays were performed to investigate antioxidant-related pathways. Additionally, *in vitro* gastrointestinal digestion of oils and cell-based assays (MCD4 cells) were conducted to evaluate antioxidant activity, cell viability, and reactive oxygen species (ROS) modulation. Olive leaf extracts were also tested for their bioactive properties.

**Results:**

The nutraceutical milling process significantly enhanced the retention of bioactive compounds, increasing total phenols and tocopherols in both cultivars. Bambina oils exhibited antioxidant profiles comparable to those of Coratina. Gene expression and enzymatic analyses revealed cultivar-specific regulation of antioxidant pathways, with Bambina showing enhanced activation of lipoxygenase-related mechanisms. *In vitro* experiments on renal collecting duct cells (MCD4) revealed that the antioxidant characteristics of both olive leaf extracts and digested oils from Bambina and Coratina cultivars were comparable.

**Conclusion:**

The Bambina cultivar shows significant potential as a nutraceutical olive variety, combining resilience to environmental stresses with high-quality bioactive profile. The application of advanced low-temperature milling further enhances its nutritional value. These findings support the valorization of Bambina olive oil as a competitive and health-promoting product in the global market.

## 1 Introduction

*Olea europaea* (olive tree) is one of the oldest and most economically significant crops worldwide, with cultivation predominantly concentrated in the Mediterranean basin. The species is believed to have originated in Asia Minor and the Levant, where it was first domesticated between 6,000 and 8,000 years ago. Today, approximately 95% of global olive production occurs in the Mediterranean region. Belonging to the *Oleaceae* family, which includes about 600 species, the olive tree is the only species cultivated primarily for food purposes (Muzzalupo, 2012).

In Italy, Apulia region stands out as the foremost olive oil-producing region, hosting approximately 50 million olive trees thanks to its highly favourable pedoclimatic conditions (ISMEA; Acciani et al., 2015). Although the outbreak of the pathogenic bacterium *Xylella fastidiosa* has severely affected many olive groves particularly in the southern part of the region since 2013, Apulia continues to retain its leading role in olive oil production, showing its remarkable resilience. The region is distinguished by a rich and diverse olive germplasm (Sion et al., 2019; Miazzi et al., 2020), exhibiting unique nutritional profiles and biochemical properties shaped by its specific pedo-climatic and agricultural conditions (Pascual et al., 2013; Sardaro et al., 2016). Among the autochthonous cultivars, the Coratina variety is particularly notable for its high phenolic content, which imparts a characteristic pungent and bitter taste typical of some virgin olive oils (Caponio et al., 1999; Rotondi et al., 2010). This pungency is primarily due to elevated levels of bioactive compounds such as oleuropein derivatives, hydroxytyrosol, and tyrosol, which possess antioxidant properties relevant to modulating inflammatory processes (Cariello et al., 2020; Difonzo et al., 2017). In contrast, the Bambina cultivar is appreciated for its mild and balanced flavour, a characteristic reflected in its traditional name, which means ‘child’, because supposed to be more appreciated by children according to the oral tradition of elderly. This milder sensory profile is related to its low concentration of oleocanthal, a compound responsible for pungency, although the significant content of phenols (Squeo et al., 2019). Moreover, *cv* Bambina is characterized by a good nutritional profile, thanks to an appreciable concentration of vitamin E and a well-balanced fatty acid composition rich in oleic acid, which enhance its antioxidant capacity and contribute to its relevance as a source of bioactive molecules with potential health benefits (Baiano et al., 2013; Sabetta et al., 2021). High antioxidant levels significantly influence both the sensory quality and shelf-life of olive oil, as lipid oxidation represents the primary pathway for oil deterioration. Virgin olive oil is distinctive among food products because it is produced without chemical treatments. To be classified as extra virgin olive oil (EVOO), the oil must satisfy stringent chemical and sensory criteria, which are affected by factors such as the olive cultivar, geographic origin, and climatic conditions. Furthermore, technological variables, including extraction methods, storage conditions, and distribution parameters, critically impact oil quality and classification (Caipo et al., 2021; Krichene et al., 2010). In this context, recent efforts have been directed towards the development of advanced processing methods, such as the nutraceutical milling technique. This method represents a modified olive oil extraction process specifically designed to preserve and enhance the concentration of bioactive compounds (phenols, tocopherols, and other antioxidants) by maintaining low temperatures throughout all stages of olive paste processing and oil extraction. This approach minimizes oxidative degradation and enzymatic activity losses, thereby yielding oils with superior nutritional, functional, and sensory qualities (Gagour et al., 2024).

The chemical composition of olive oil is predominantly determined by volatile and phenolic compounds, which govern its oxidative stability, flavour, and health benefits (Del Carlo et al., 2004; Gómez-Caravaca et al., 2016). Polyphenols, such as tyrosol and oleuropein are also abundant in olive leaf extracts, promoting their use as natural food protectants (Difonzo et al., 2017; Rahmanian et al., 2015). Another key antioxidant compound is alpha-tocopherol, a major lipophilic antioxidant and member of the vitamin E family; its concentration is influenced by both the specific olive cultivar and the developmental stage of fruit ripening (Tura et al., 2007; Jukić Špika et al., 2015). In this context, the characterization of olive germplasm is essential for its preservation and valorisation, representing a strategic advantage in the face of global market competition.

An additional strength of *cv* Bambina is represented by its notable rusticity, that describes its ability to adapt and thrive under adverse environmental or agricultural conditions, without requiring intensive agronomic management. Indeed, *cv* Bambina is either well adapted to poor or calcareous soils, which are widespread in many areas of Apulia, or able to grow in hilly or semi-arid regions with limited water availability. All these features, together with its good nutritional profile, makes *cv* Bambina an excellent candidate to be promoted among the autochthonous Apulian olive tree varieties.

The present study aims to provide a comprehensive characterization of the Bambina olive cultivar by analysing drupes, leaves, and derived products, including pastes and oil. It also evaluates the impact of advanced oil extraction methods on the retention of bioactive compounds and the nutritional quality of oils. A comparative approach was adopted with the widely cultivated Coratina variety to evaluate the nutraceutical potential of *cv* Bambina. Additionally, *in vitro* studies using renal collecting duct cells were conducted to investigate the antioxidant properties of olive leaf extract and digested oils, focusing on vitamin E and other bioactive compounds.

## 2 Materials and methods

### 2.1 Plant material

Drupes and leaves samples were collected from individual trees of the cultivars Coratina and Bambina, grown in the “A. Raguso” groves (Gravina in Puglia, Bari, Southern Italy; 40°49’00” N, 16°25’00” E). Drupes were specifically harvested at the beginning of ripening. Samples were randomly collected from the tree canopies, immediately frozen in liquid nitrogen and stored at −80 °C until analysis. Three biological replicates were collected for each cultivar, and all experiments were performed in triplicates.

### 2.2 Milling

**Figure 1:**
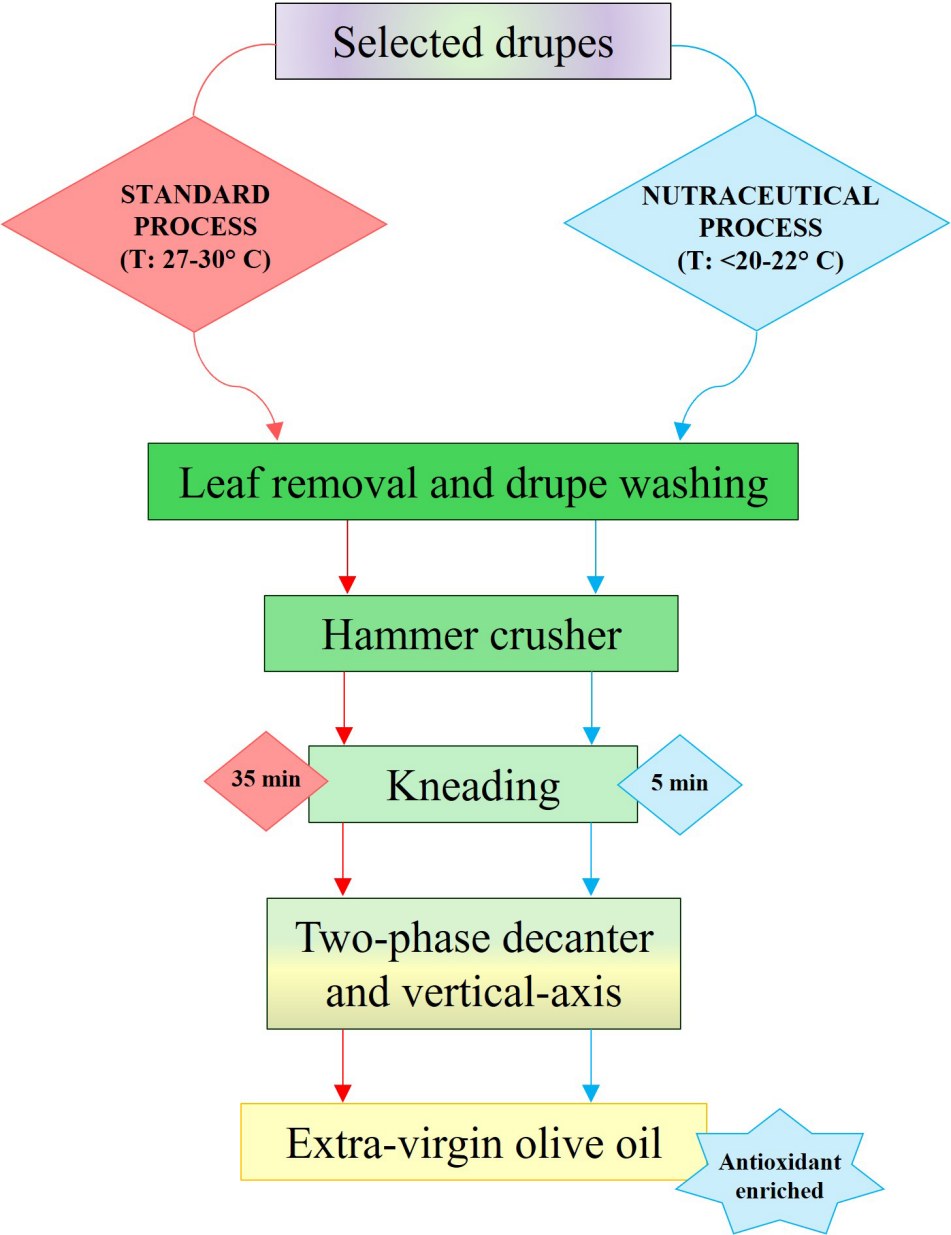
Flowchart of olive oil extraction comparing standard vs. nutraceutical processes.

The harvested drupes were carefully selected using an optical sorter; those with maturity index (MI) less than 2 (Guzmán et al., 2015) were processed, as shown in **Figure 1**, within three hours of harvesting. In particular, two different milling approaches were compared: a standard method and a low-temperature milling process (here called “nutraceutical”). The main differences consisted principally in the application of controlled low temperatures (< 22 °C) throughout all stages of olive processing and oil extraction, and in a significant reduction of the kneading times (5 min *vs* 35 min) with the aim to increase phenolic compounds and vitamins in the extra virgin olive oils. The described method is subject to licensing (n. 102025000025843/2025). Each milling process was independently carried out three times (n = 3).

### 2.3 RNA isolation and transcription

Frozen mesocarp and pastes were homogenized using a tissue lyser (Qiagen, Chatsworth, CA, USA). Total RNA was extracted from 150 mg of powdered sample using the RNeasy Plant Mini Kit (Qiagen, Chatsworth, CA, USA) followed by purification with 8 M lithium chloride (Sigma-Aldrich, St. Louis, MO, USA) as described in Sabetta et al. (2022). RNA quality was assessed using a Nanodrop 2000C spectrophotometer (Thermo Scientific, Waltham, MA, USA), and integrity was verified by electrophoresis on a 1% agarose gel. cDNA was synthesized from 1,7 μg of total RNA using the QuantiTect Reverse Transcription Kit (Qiagen, Chatsworth, CA, USA) according to the manufacturer’s instructions.

### 2.4 Gene expression analysis

For quantitative real-time PCR (RT-qPCR), 1 μL of 10-fold diluted cDNA was used in each reaction. Previously published oligonucleotide primers were selected for gene expression analysis of eight candidate genes (*OeGGR*, *OeHPPD*, *OeHGGT*, *OeVTE1*, *OeVTE2*, *OeVTE3*, *OeVTE4*, and *OeVTE5*) involved in the tocopherol biosynthetic pathway, plus six genes implicated in antioxidant processes during drupe maturation (*OePOD*, *OePOD42like*, *OePPO1*, *OePPO3*, *OeLOX*, *Oe2LOX2*) (**Supplementary Table 1**). Only for the *OePOD* gene specific primer pair was designed, using the coding sequence (CDS) deposited in the NCBI database (http://www.ncbi.nlm.nih.gov/). The *OeEF1α* gene (accession n. AM946404) was used as reference gene. Calibration curves were established using 1:5 serial dilution of cDNA to determine the amplification efficiency of each primer pairs. Reactions were performed in triplicate, using the Sso Advanced Universal SYBR^®^ Green Supermix (Bio-Rad Laboratories, Hercules, CA, USA) on a CFX96 Touch Real-Time PCR Detection System (Bio-Rad Laboratories, Hercules, CA, USA). Thermal cycling conditions were as follows: hot start at 95 °C for 3 minutes, followed by 40 amplification cycles (95 °C for 10 s, 60 °C for 30 s). Amplification specificity was verified by melting curve analysis. Relative transcript levels were calculated using the 2^−ΔΔCt^ method, as described by Livak and Schmittgen (2001).

### 2.5 Enzymatic activities in olive pastes

Enzymatic activities were determined on acetone powders prepared as reported by García-Rodríguez et al. (2011), with minor modifications. Briefly, samples were weighed and then homogenized with pre-chilled acetone. The homogenate was filtered, re-homogenized twice with cold acetone, and subsequently washed with diethyl ether. Finally, the samples were dried under a fume hood at room temperature and stored at −20 °C until further analysis. Prior to enzymatic assays, samples were accurately weighed to normalize enzymatic activities to fresh weight. Peroxidase (POD) and polyphenol oxidase (PPO) activities were measured according to the protocol of Peres et al. (2016). One unit (U) of POD activity was formulated as the amount of enzyme that catalyzes the oxidation of 1 μmol guaiacol min^-1^ mL^-1^ of enzyme extract, based on a molar extinction coefficient (e) of 26.6 mM^-1^ cm^-1^. PPO activity was evaluated using 30 mM catechol as substrate, measuring the increase in absorbance at 420 nm after 5 min incubation at 25 °C. One unit of PPO activity was formulated as the amount of enzyme that caused a change in absorbance of 0.001 min^-1^ mL^-1^ at 25 °C. Lipoxygenase (LOX) activity was assessed based on the formation of conjugated dienes, as described by Axelrod et al. (1981). One unit of LOX was defined as the amount of enzyme that catalyses the formation of 1 μmol of conjugated diene product per minute, measured at 234 nm, using an extinction coefficient (ϵ) of 25000 M^-^¹ cm^-^¹.

### 2.6 Chemical characterization of monovarietal oils

Phenol and tocopherol characterization were carried out as described in Zago et al. (2019). In particular, the total phenol content (TPC) of the extra virgin olive oils was measured following the Folin-Ciocâlteu colorimetric assay. The quantitation was performed using an external calibration curve of gallic acid (GAE) and expressed as mg of GAE kg^-1^ of oil.

The determination of the single phenolic compounds was carried out by HPLC. The extraction was performed using 5 g of oil mixed with 250 μL of 100 mg kg^-1^ gallic acid solution (internal standard), 1 mL of hexane and 2 mL of methanol/water mixture (70:30 v/v).

After extraction, an aliquot of the methanolic phase (250 μL) was transferred to a HPLC vial with 250 μL of water/acetic acid (99:1 *v*/v). UHPLC binary system (Dionex Ultimate 3000 RSLC, Waltham, MA, USA) equipped with a quaternary pump (HPG 3200RS), auto-sampler (WPS 3000), stationary phase compartment (TCC 3000), diode array detector (3000 RS) and the Chromeleon software for data acquisition and processing was used. The stationary phase was an Acclaim C18 analytical column (150 × 4.6 mm i.d., 3 μm) (Thermo Scientific, Waltham, MA, USA). The mobile phases were (A) water/acetic acid (99:1 v/v) and (B) methanol/acetonitrile/acetic acid (50:49:1 v/v/v) at a constant flow rate of 1 mL/min. The column temperature was set at 30 °C. Diode array detection was monitored at 280 nm, and spectra were recorded at a wavelength range 200–380 nm. The identification of phenolic compounds was performed by comparing the peak retention times with those obtained by the injection of pure standards and with COI reference method (COI/T.20/Doc. No 29/Rev.2) (COI, 2022). The quantification was achieved using a gallic acid internal standard. The results are expressed as mg of gallic acid equivalents (GAE) kg^-1^ of oil.

The determination of the tocopherols was performed using the same UHPLC apparatus and stationary phase. Approximately 0.10 g of oil was dissolved in 1 mL of 2-propanol for HPLC, then the mixture was vortexed, and filtered through PTFE filters (0.45 μm). The mobile phase consists of a mixture of acetonitrile and methanol (1:1 *v*/v) at a constant flow rate of 1 mL min^-1^ in isocratic elution. The injection volume was 20 μL. The quantification of tocopherols was reached by means of FLD detector (Dionex 3400RS, Waltham, MA, USA), set at an excitation wavelength of 295 nm and an emission at 325 nm. The quantification was performed using external calibration curves (α-tocopherol and the sum of β- and γ-tocopherols), expressing the results as mg kg^-1^ of tocopherols.

### 2.7 *In vitro* gastrointestinal digestion of oil samples

A simulation of *in vitro* gastrointestinal digestion of the oil was performed following Caponio et al. (2025a) using a two-phase model. First, gastric digestion was simulated by incubating the samples with pepsin and hydrochloric acid (HCl) for 3 hours at 37 °C. Subsequently, intestinal digestion was simulated by incubating the samples with pancreatin and bile salts for an additional 3 hours at the same temperature to mimic small intestinal conditions. In brief, 10 mL of each oil sample were mixed with 10 mL of an α-amylase solution (56 mg/ml; Sigma-Aldrich Chemistry, St. Louis, MO, USA) and 10 mL of a pepsin solution containing NaCl (125 mM L^-1^), KCl (7 mM L^-1^), NaHCO_3_ (45 mM L^-1^) and pepsin (3 g L^-1^) (all from Sigma-Aldrich Chemistry, St. Louis, MO, USA). The pH was adjusted to 2 with hydrochloric acid and the mixture incubated at 37 °C for 180 minutes in a shaking water bath. At the end of the gastric phase, the digests were mixed at a ratio of 1:1 with an intestinal solution containing pancreatin (0.1 g 100–^1^ mL; Sigma-Aldrich, St. Louis, MO, USA) and bile salts (0.15 g 100–^1^ mL; Oxoid™, Hampshire, UK). The pH was then raised to 8 using NaOH and the samples were incubated for a further 180 minutes at 37 °C under constant agitation. Samples were collected at the end of the duodenal phase (DP). To stop pancreatic activity, DP samples were acidified to pH 2 using 37% HCl, then neutralised to pH 8 with 35% NaOH. The digested mixtures were then centrifuged at 18,500 × g for 20 minutes and the upper oil layer removed. The aqueous micellar fraction was then filtered through 0.2 µm cellulose acetate membranes to remove residual turbidity, after which it was stored at -80 °C until analysis.

### 2.8 *In vitro* assays of digested oil on cell cultures

#### 2.8.1 Cell culture and treatments

Renal collecting duct cells (MCD4) were cultured as already reported (Ranieri et al, 2019). Briefly, cells were grown in Dulbecco’s Modified Eagle’s Medium (DMEM/F12) supplemented with 5% fetal bovine serum, 2 mM L-glutamine, 100 i.u. mL^-1^ penicillin, 100 μg mL^-1^ streptomycin, and 5 μM dexamethasone at 37 °C, 5% CO_2_.

#### 2.8.2 Calcein-AM cell viability assay and reactive oxygen species detection

Cell viability was assessed using the method described by Caponio et al. (2025b), with some modifications. Cells were seeded into 96-well culture plates and allowed to grow until they reached approximately 90% confluence. The cells were then either maintained under basal conditions (untreated control, CTR-) or exposed to digested oil extracts at increasing dilutions (1:200, 1:150, 1:100 and 1:50) for 24 hours. After this time, cell viability was determined using the calcein-AM assay. The cells were incubated with calcein-AM (1 µM) for 45 minutes at 37 °C. Fluorescence intensity was then recorded using a FLUOstar Omega fluorimeter (software version 5.10 R2, BMG LABTECH, Offenburg, Germany). Measurements were performed at excitation and emission wavelengths of 508 nm and 529 nm, respectively.

Intracellular ROS levels were quantified as previously described (Caponio et al., 2024). Following the treatments, the cells were incubated with 10 μM dihydrorhodamine-123 at 37 °C for 30 minutes, after which they were given a further 30-minute recovery period in complete culture medium. The cells were then lysed using RIPA buffer, which contained 150 mM NaCl, 10 mM Tris-HCl (pH 7.2), 0.1% SDS, 1.0% Triton X-100, 1% sodium deoxycholate and 5 mM EDTA. The cell lysates were then clarified by centrifugation at 12,000× g for 10 minutes at 4 °C and the resulting supernatant was collected for ROS analysis. Cells treated with tert-butyl hydroperoxide (tBHP, 2 mM for 30 minutes) were included as a positive control. Fluorescence was measured using a FLUOstar Omega microplate reader (BMG LABTECH, Offenburg, Germany) with an excitation wavelength of 508 nm and an emission wavelength of 529 nm.

### 2.9 *In vitro* assays of olive leaf extract on cell cultures

#### 2.9.1 Olive leaf extract preparation

Olive leaf extract was prepared according to Caponio et al. (2022), with some modifications. Briefly, 3 g of olive leaf were mixed with water (1:10 w/v), vortexed for 10 min, sonicated for 15 min (Elmasonic S 60 H, ELMA, Singen, Germany), and finally centrifugated at 12,000× g for 10 min (SL 16R Centrifuge, Thermo Scientific, Waltham, MA, USA) to recover the extract. Extractions were repeated twice with 30 mL of water. The three extracts were combined, filtered as above reported, and stored at -20 °C until analysis. All extracts were prepared in triplicate.

#### 2.9.2 Total phenol content determination

Olive leaf extract was evaluated for the total phenol content (TPC) according to the Folin–Ciocalteu method following the procedure in section 2.6 with some modifications, as reported in Caponio et al. (2022). Briefly, 980 μL of H_2_O Milli-Q, 20 μL of appropriately diluted extract, and 100 μL of Folin–Ciocalteu reagent were added. After 3 min, 800 μL of 7.5% Na_2_CO_3_ were added, and then the sample was stored in the dark for 60 min. The absorbance was read at 720 nm using an Evolution 60s UV-visible spectrophotometer (Thermo Fisher Scientific, Rodano, Italy). The quantitation was performed using an external calibration curve of gallic acid (GAE) and expressed as µg of GAE mL^-1^ of extracts.

#### 2.9.3 Cell assays

MCD4 cell line was cultured as described in section 2.8.1. Briefly, cells were left under basal condition (untreated, CTR-), or treated with olive leaf extract (0.1, 0.5, 1.0, 5.0, 10.0, 20.0 μg mL^-1^ of TPC) for 24 h. Calcein-AM assay and ROS detection were then performed following the procedure descripted in section 2.8.2.

### 2.10 Statistical analysis

Experimental data are presented as mean ± standard deviation of three biological replicates. As reported by Nunes et al. (2015), the mean value of triplicate measurements is the best estimate of the analytes/parameters in the sample as well as the standard deviation can be considered as the best estimate of the experimental error. Differences between groups were assessed using Student’s *t*-test or one-way ANOVA, followed by appropriate *post-hoc* tests, using GraphPad software (San Diego, CA, USA). Significance levels are indicated as follows: *p* < 0.05 (*), *p* < 0.01 (**), *p* < 0.001 (***) and *p* < 0.0001 (****).

## 3 Results

### 3.1 Milling and chemical characterization of monovarietal oils

In the nutraceutical milling method (**Figure 1**), all steps of the olive oil extraction process, from the orchard to the mill, were carried out at temperatures below 20-22 °C, compared with the higher temperatures (27-30 °C) employed in the standard procedure. Harvested drupes were immediately stored and transported in cooled containers. Both protocols included leaf removal and drupe washing, followed by hammer crushing. To minimize oxidation and peroxide formation, the kneading time was reduced from 35 to 5 min, thereby preserving antioxidant compounds without impairing the release of flavour compound or oil aroma. These process modifications yielded an extra virgin olive oil enriched in antioxidant compounds. Specifically, both total phenol and tocopherol contents were significantly higher in nutraceutical Coratina and Bambina oils (hereafter referred to as CN and BN, respectively) than in their standard counterparts (CS and BS), with increases of approximately 67% and 33% in total phenols and 22% and 8% in tocopherols for CN and BN oils, respectively (**Table 1**). Individual phenolic compounds exhibited trends consistent with those observed for total phenols. Hydroxytyrosol, 3,4-DHPEA-EDA, pinoresinol, 3,4-DHPEA-EA, apigenin, and p-HPEA-EA were the most affected, whereas syringic acid and p-coumaric acid did not differ significantly between treatments. Other phenolic compounds showed variable trends. HPLC analysis further confirmed a significant increase in total phenolic content achieved through the nutraceutical processing method (**Table 1**).

**Table 1:** Total Tocopherols, Total Phenolic Compounds (TPC) and the main phenolic compounds of standard Coratina (CS), nutraceutical Coratina (CN), standard Bambina (BS) and nutraceutical Bambina (BN) oils.

Parameters	Samples			
	CS	CN	BS	BN
Total Tocopherols (mg/kg^-1^)	314.05 ± 8.25^c^	383.34 ± 12.01^a^	323.97 ± 4.70^c^	349.83 ± 5.03^b^
TPC (mg GA kg^-1^)	333.67 ± 14.70^c^	558.70 ± 14.16^a^	323.70 ± 7.69^c^	431.42 ± 35.49^b^
*Phenolic compounds (mg kg^-1^)*				
Hydroxytyrosol	0.69 ± 0.12^c^	1.60 ± 0.22^b^	0.40 ± 0.05^c^	2.45 ± 0.40^a^
Tyrosol	2.70 ± 0.07^a^	3.83 ± 0.52^a^	1.33 ± 0.04^b^	3.09 ± 0.63^a^
Vanillic acid	0.86 ± 0.08^a^	0.28 ± 0.07^b^	0.35 ± 0.04^b^	0.34 ± 0.04^b^
Syringic acid	0.18 ± 0.03^a^	0.16 ± 0.05^a^	0.21 ± 0.02^a^	0.26 ± 0.04^a^
p-Coumaric acid	0.81 ± 0.06^a^	0.66 ± 0.21^a^	0.85 ± 0.10^a^	0.56 ± 0.05^a^
3,4-DHPEA-EDA	20.65 ± 0.78^b^	24.24 ± 1.11^c^	13.09 ± 0.58^a^	18.17 ± 1.17^b^
p-HPEA-EDA	11.71 ± 0.14^c^	13.38 ± 0.08^b^	15.96 ± 0.14^a^	10.84 ± 0.65^c^
Pinoresinol	6.27 ± 0.13^d^	15.01 ± 0.59^a^	8.38 ± 0.19^c^	10.64 ± 0.23^b^
Luteolin	6.38 ± 0.08^b^	9.53 ± 0.28^a^	6.98 ± 0.16^b^	6.96 ± 0.41^b^
3,4-DHPEA-EA	9.64 ± 0.20^c^	19.98 ± 0.34^a^	8.55 ± 0.24^c^	13.00 ± 0.84^b^
Apigenin	3.73 ± 0.09^c^	15.80 ± 0.62^a^	3.48 ± 0.34^c^	9.39 ± 0.86^b^
p-HPEA-EA	3.85 ± 0.18^c^	10.76 ± 0.31^a^	3.70 ± 0.55^c^	6.31 ± 0.52^b^
*Total*	*67.48 ± 1.15^c^*	*114.89 ± 2.54^a^*	*63.29 ± 1.34^c^*	*82.62 ± 4.10^b^*

Data are reported as mean ± SD. Different letters within the same row indicate statistically significant differences between treatments according to one-way ANOVA followed by Tukey’s HSD test (*p* < 0.05).

### 3.2 Candidate gene selection and expression analysis

Tocochromanols (vitamin E), which include both tocopherols and tocotrienols, are among the most potent antioxidant compounds in virgin olive oil (Aggarwal et al., 2010). Accordingly, candidate genes involved in tocopherol biosynthesis were selected to assess the antioxidant potential of *cv* Bambina in comparison with the well-characterized *cv* Coratina. In addition, genes encoding antioxidant enzymes, such as members of the PPO, POD and LOX families, which play key roles in oxidative stress responses and in the determination of oil quality, were included in the analysis. These investigations were conducted on both drupes and olive pastes obtained from the two milling strategies.

#### 3.2.1 In drupes

**Figure 2:**
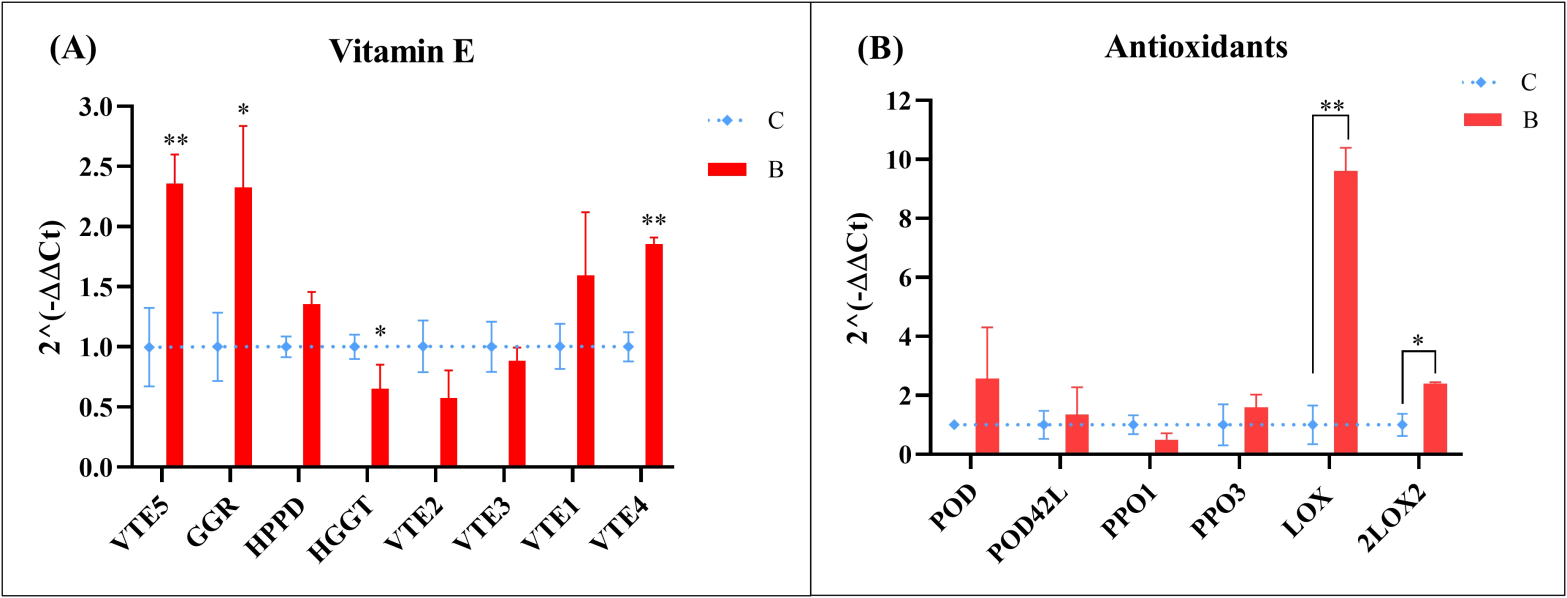
Relative expression levels of genes involved in **(A)** vitamin E biosynthesis and **(B)** antioxidant activity in drupes of Bambina and Coratina cultivars. Data represent the mean values over two years. Statistical analysis was performed using two-tailed Student’s *t*-test. Asterisks denote statistically significant differences: * *p* < 0.05, ** *p* < 0.01, *** *p* < 0.001. C, Coratina; B, Bambina.

To investigate the regulation of tocochromanol biosynthesis, the expression levels of eight key genes involved in vitamin E production were quantified in drupes of *cv* Bambina and *cv* Coratina (**Figure 2A**), namely *OeVTE5*, *OeGGR*, *OeHPPD*, *OeHGGT*, *OeVTE2*, *OeVTE3*, *OeVTE1*, and *OeVTE4*. Of these, five genes were upregulated in *cv* Bambina relative to *cv* Coratina, with statistically significant differences observed for *OeVTE5*, *OeGGR* and *OeVTE4*, which displayed approximately 2-fold higher transcript levels in *cv* Bambina.

To identify candidate genes potentially influencing drupe antioxidant capacity, the literature on olive POD, PPO and LOX gene families was reviewed. Given the large number of family members, the analysis was restricted to those genes reported as predominantly expressed during the fruit breaker stage and subsequent ripening phases, namely *OePOD*, *OePOD42like*, *OePPO1*, *OePPO3*, *OeLOX* and *Oe2LOX2*. With the exception of *OePPO1*, all examined genes showed a trend toward higher expression in *cv* Bambina, although differences were statistically significant only for the two LOX family members (**Figure 2B**). Specifically, *OeLOX* exhibited the most pronounced upregulation, with transcript levels approximately 9-fold higher in *cv* Bambina than in *cv* Coratina (*p* < 0.01), while *Oe2LOX2* also showed a statistically significant increase (*p* < 0.05), collectively suggesting the activation of the lipoxygenase pathway in this cultivar. This expression pattern was reproducible across multiple sampling years, indicating a cultivar-specific regulation of antioxidant-related gene pathways.

#### 3.2.2 In olive pastes

**Figure 3:**
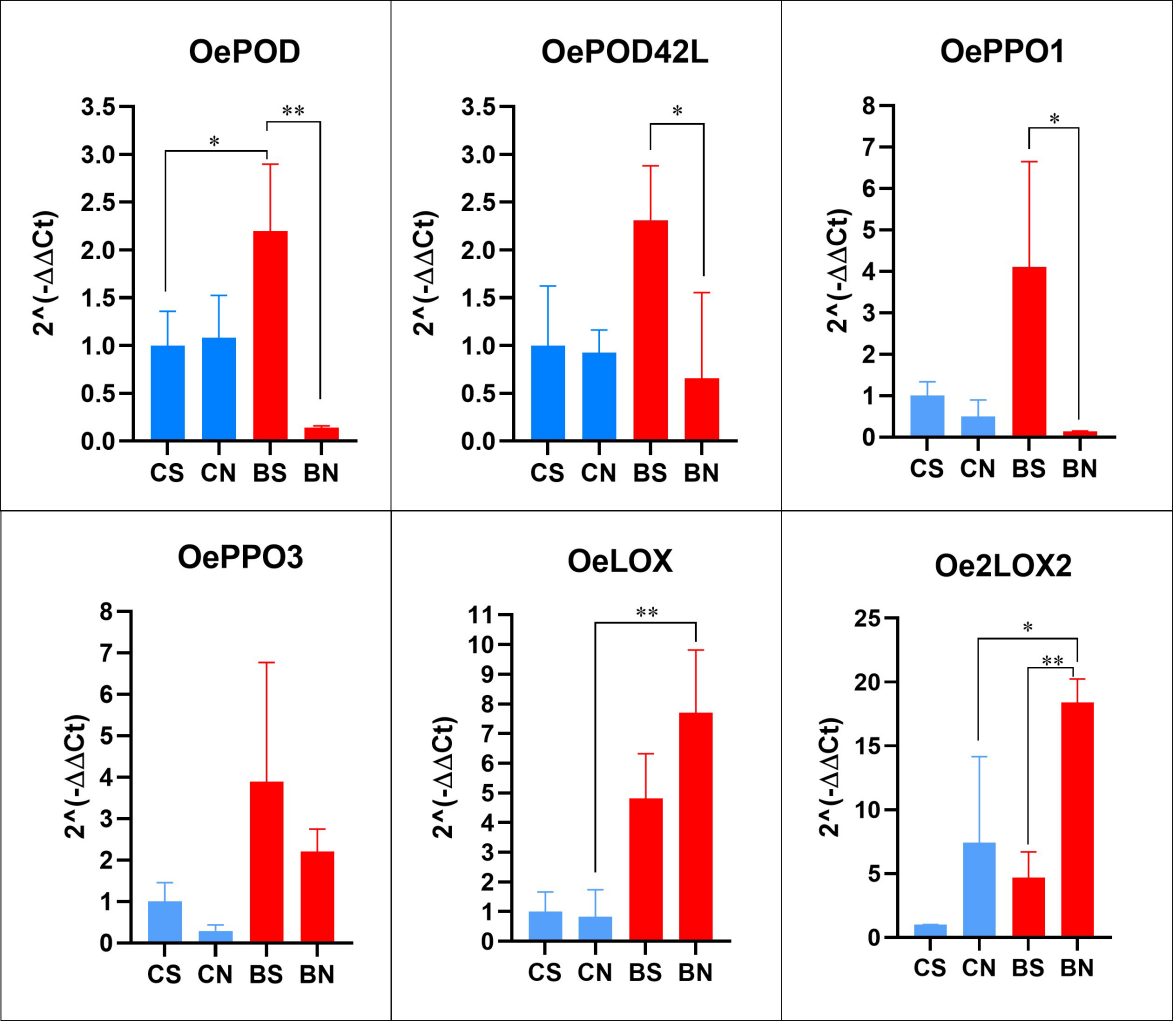
Relative expression levels of antioxidant related-genes in olive pastes from standard and nutraceutical milling of Coratina and Bambina drupes. Data were analysed using one-way ANOVA. Asterisks denote statistically significant differences: * *p* < 0.05, ** *p* < 0.01, *** *p* < 0.001. CS: Coratina standard, CN: Coratina nutraceutical, BS: Bambina standard, BN: Bambina nutraceutical.

To evaluate the impact of milling strategy on antioxidant gene expression, transcript levels of the same set of genes analysed in Coratina and Bambina drupes were quantified in their corresponding standard (CS and BS) and nutraceutical (CN and BN) pastes (**Figure 3**). Nutraceutical milling induced opposite transcriptional responses depending on the gene family considered. *OePOD*, *OePOD42L*, and *OePPO1* were significantly downregulated in nutraceutical compared to standard pastes of *cv* Bambina (*p* < 0.05 and *p* < 0.01, respectively), while no significant effect of milling condition was observed in *cv* Coratina. Conversely, both lipoxygenase members were markedly upregulated under nutraceutical conditions, with *OeLOX* and *Oe2LOX2* reaching significantly higher transcript levels in BN relative to all other conditions (*p* < 0.01). Notably, *Oe2LOX2* expression in BN was approximately 4-fold higher than in BS and CN and nearly 19-fold higher than in CS, representing the highest expression level recorded across all samples. These results indicate that nutraceutical milling selectively promotes *LOX* pathway activation while suppressing *POD* and *PPO* transcription specifically in *cv* Bambina, a pattern consistent with enhanced aromatic precursor biosynthesis and reduced oxidative enzyme activity in this cultivar.

### 3.3 Antioxidant enzymatic activity in olive pastes

**Figure 4:**
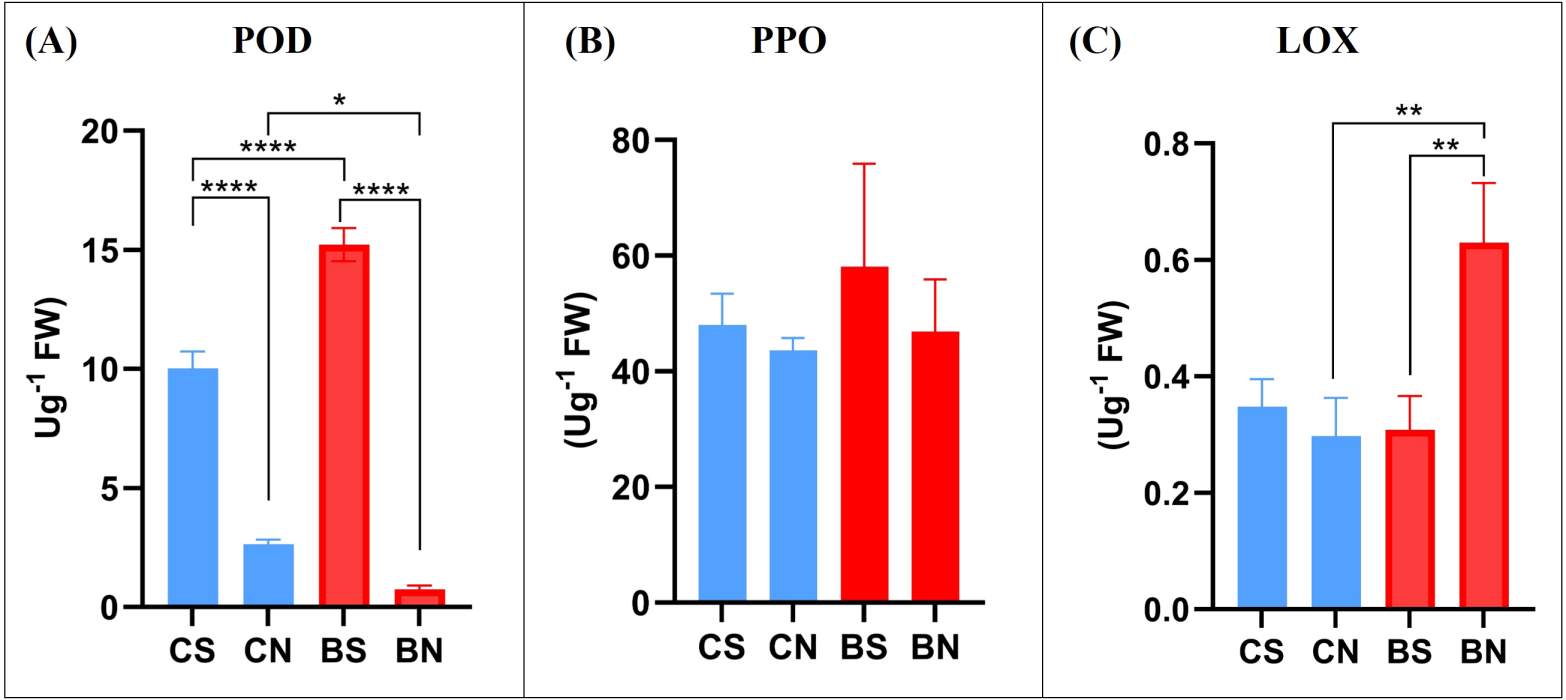
Mean values of the enzymatic activity of **(A)** POD, **(B)** PPO and **(C)** LOX in olive pastes of Coratina and Bambina cultivars in the standard and nutraceutical milling. A one-way ANOVA test was performed (* *p* < 0.05, ** *p* < 0.01, *** *p* < 0.001, **** *p* < 0.0001). CS: Coratina standard, CN: Coratina nutraceutical, BS: Bambina standard, BN: Bambina nutraceutical.

To corroborate the gene expression results, the activity of POD, PPO and LOX enzymes was assessed in standard and nutraceutical pastes of *cv* Coratina and *cv* Bambina (**Figure 4**). Consistent with the observed transcriptional patterns, the nutraceutical milling significantly decreased POD activity in olive pastes of both cultivars, while LOX activity was markedly enhanced exclusively in *cv* Bambina (**Figures 4A, C**). This cultivar-specific enzymatic response mirrors the gene expression results and further supports a differential regulation of antioxidant pathways between the two cultivars. The higher LOX activity observed in BN pastes suggests a greater capacity for the synthesis of C6 volatile compounds derived from the lipoxygenase pathway, which typically confer desirable green and fruity sensory attributes to virgin olive oil. In contrast, PPO activity showed no statistically significant differences between cultivars or milling conditions (**Figure 4B**).

### 3.4 Cell viability and ROS detection of digested oil samples

The effects of Bambina and Coratina digested oils (B-ol and C-ol) at different dilutions on cell viability and ROS content in MCD4 cells were characterized in standard and nutraceutical conditions (**Table 2**). Interestingly, the incubation with BS and BN samples yielded dilution-dependent effects. In particular, BS showed high values up to 1:100 dilution (0.90 ± 0.06), with a significant reduction at 1:50 dilution (0.37 ± 0.08). BN maintained relatively high values up to 1:150 dilution (0.91 ± 0.08), but similarly declined at 1:50 dilution (0.35 ± 0.07). Comparable behaviours were observed for CS and CN samples, both with high and stable values up to 1:100 dilution with significant decreases at 1:50. Based on these results, a 1:150 dilution was selected for subsequent experiments.

**Table 2:** Cell viability of digested oil samples.

Dilution	CTR-	CS	CN	BS	BN
*1:200*	1.00 ± 0.00^a^	0.72 ± 0.08^c^	0.91 ± 0.03^b^	0.86 ± 0.04^b^	0.77 ± 0.08^c^
*1:150*	1.00 ± 0.00^a^	0.88 ± 0.02^b^	0.90 ± 0.03^b^	0.87 ± 0.02^b^	0.91 ± 0.08^b^
*1:100*	1.00 ± 0.00^a^	0.83 ± 0.12^bc^	0.77 ± 0.06^c^	0.90 ± 0.06^ab^	0.81 ± 0.06^bc^
*1:50*	1.00 ± 0.00^a^	0.38 ± 0.06^c^	0.59 ± 0.13^b^	0.37 ± 0.08^c^	0.35 ± 0.07^c^

Data are reported as mean ± SD and analysed by one-way ANOVA followed by Tukey test for multiple comparisons. Data are expressed as arbitrary units (A.U.) of fluorescence intensity normalized to control cells. Different letters in row differ significantly with *p* < 0.05. CS: Coratina standard, CN: Coratina nutraceutical, BS: Bambina standard, BN: Bambina nutraceutical.

Cells were treated for 24 h at the indicated dilutions (1:200; 1:150, 1:100, 1:50). The negative control (CTR**-**; vitality: 100%) consisted of untreated cells used to evaluate basal cell viability by calcein-AM assay.

**Figure 5:**
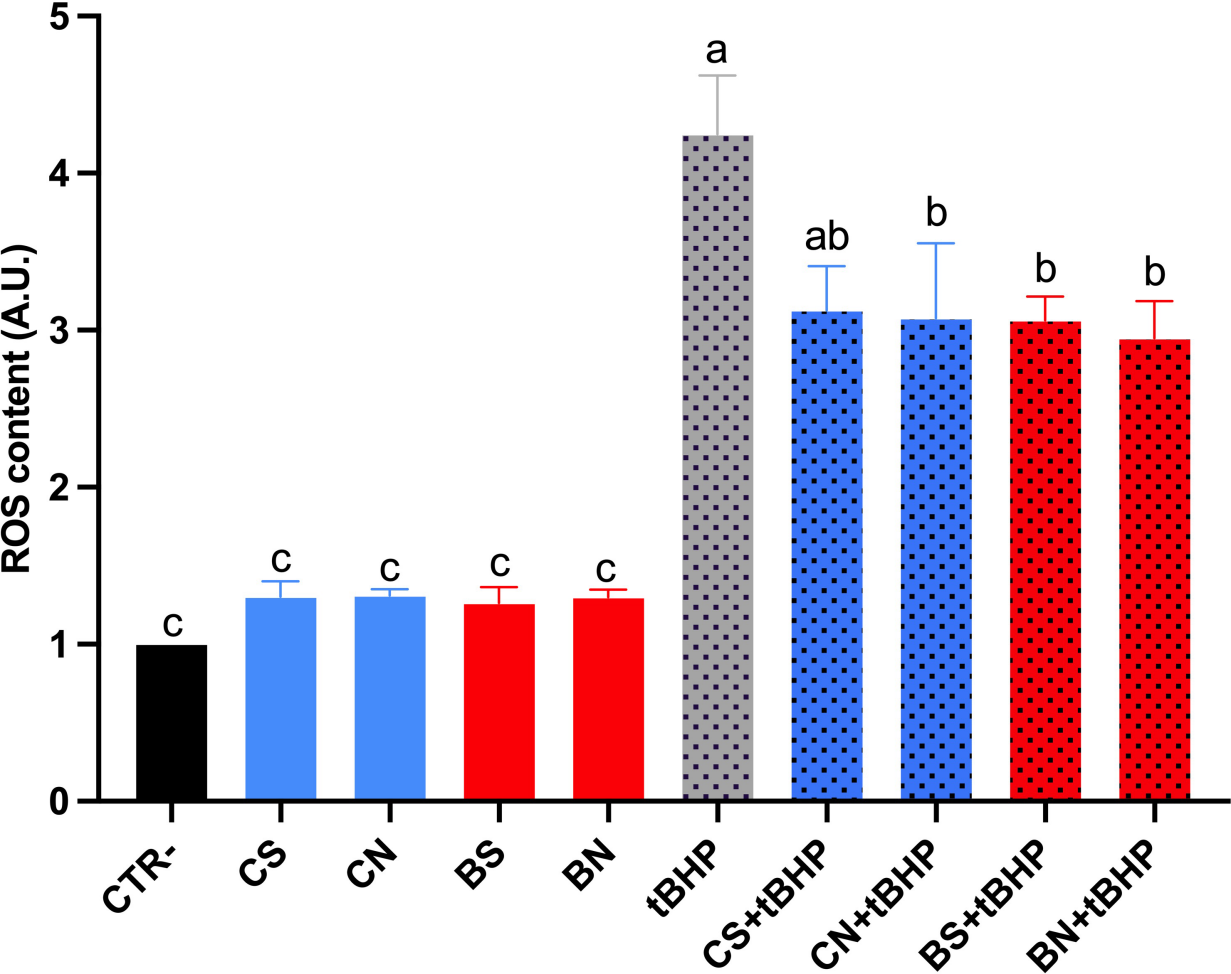
ROS content measured using dihydrorhodamine-123 fluorescence in MCD4 cells treated for 24 h at dilution of 1:150 of digested oil samples. Data are shown as mean ± SEMs and analysed by one-way ANOVA followed by Tukey’s multiple comparison test. Data are expressed as arbitrary units (A.U.) of fluorescence intensity normalized to control cells. Different letters mean a significant difference (*p ≤* 0.05) between samples. Abbreviations: CS: Coratina standard, CN: Coratina nutraceutical, BS: Bambina standard, BN: Bambina nutraceutical, tBHP, tert-butyl hydroperoxide.

ROS levels in digested samples are shown in **Figure 5**. Under basal conditions, neither cultivar’s digested oils altered the intracellular ROS, indicating no pro-oxidant action. However, in the presence of tert-butyl hydroperoxide (tBHP), a synthetic pro-oxidant compound, both Bambina and Coratina digested oils significantly reduced the tBHP-induced ROS generation, with no substantial differences between S and N samples.

### 3.5 *In vitro* effect of olive leaf extract

The effect of different concentrations of olive leaf extract on cell viability was analysed (**Table 3**). The total phenol content (TPC) of the extracts was measured for the Bambina and Coratina olive leaf varieties, yielding values of approximately 660 µg mL^-1^ and 2240 µg mL^-1^, respectively (**Supplementary Table 2**). MCD4 cells were then left untreated (CTR−) or treated with different concentrations of TPC from olive leaf extract (0.1, 0.5, 1.0, 5.0, 10.0, 20.0 μg mL^-1^) for 24 h. At the lowest concentration tested (0.1, 0.5 and 1.0 µg mL^-1^, neither B-ol nor C-ol induced a significant reduction in cell viability compared with untreated cells, suggesting that these concentrations were not cytotoxic. At higher concentration (5.0, 10 and 20 µg mL^-1^), both samples caused a reduction in cell viability. Based on these findings, a concentration of 1.0 µg mL^-1^ was selected for subsequent experiments.

**Table 3:** Cell viability of olive leaf extract samples.

Sample	CTR-	0.1 µg mL^-1^	0.5 µg mL^-1^	1 µg mL^-1^	5 µg mL^-1^	10 µg mL^-1^	20 µg mL^-1^
C-ol	1.00 ± 0.00^a^	1.02 ± 0.13^a^	0.96 ± 0.11^a^	1.01 ± 0.13^a^	0.91 ± 0.03^ab^	0.88 ± 0.02^ab^	0.80 ± 0.03^b^
B-ol	1.00 ± 0.00^ab^	1.15 ± 0.18^a^	0.99 ± 0.23^ab^	1.02 ± 0.19^ab^	0.85 ± 0.09^b^	0.84 ± 0.03^b^	0.90 ± 0.05^b^

Data are reported as mean ± SD and analysed by one-way ANOVA followed by Tukey test for multiple comparisons. Data are expressed as arbitrary units (A.U.) of fluorescence intensity normalized to control cells. Different letters in row differ significantly with *p* < 0.05. C-ol, Coratina olive leaf extract; B-ol, Bambina, olive leaf extract.

Cells were treated for 24 h at the indicated concentrations (0.1, 0.5, 1.0, 5.0, 10.0, 20.0 μg mL^-1^) of TPC. The negative control (CTR**-**; vitality: 100%) consisted of untreated cells used to evaluate basal cell viability by calcein-AM assay.

**Figure 6:**
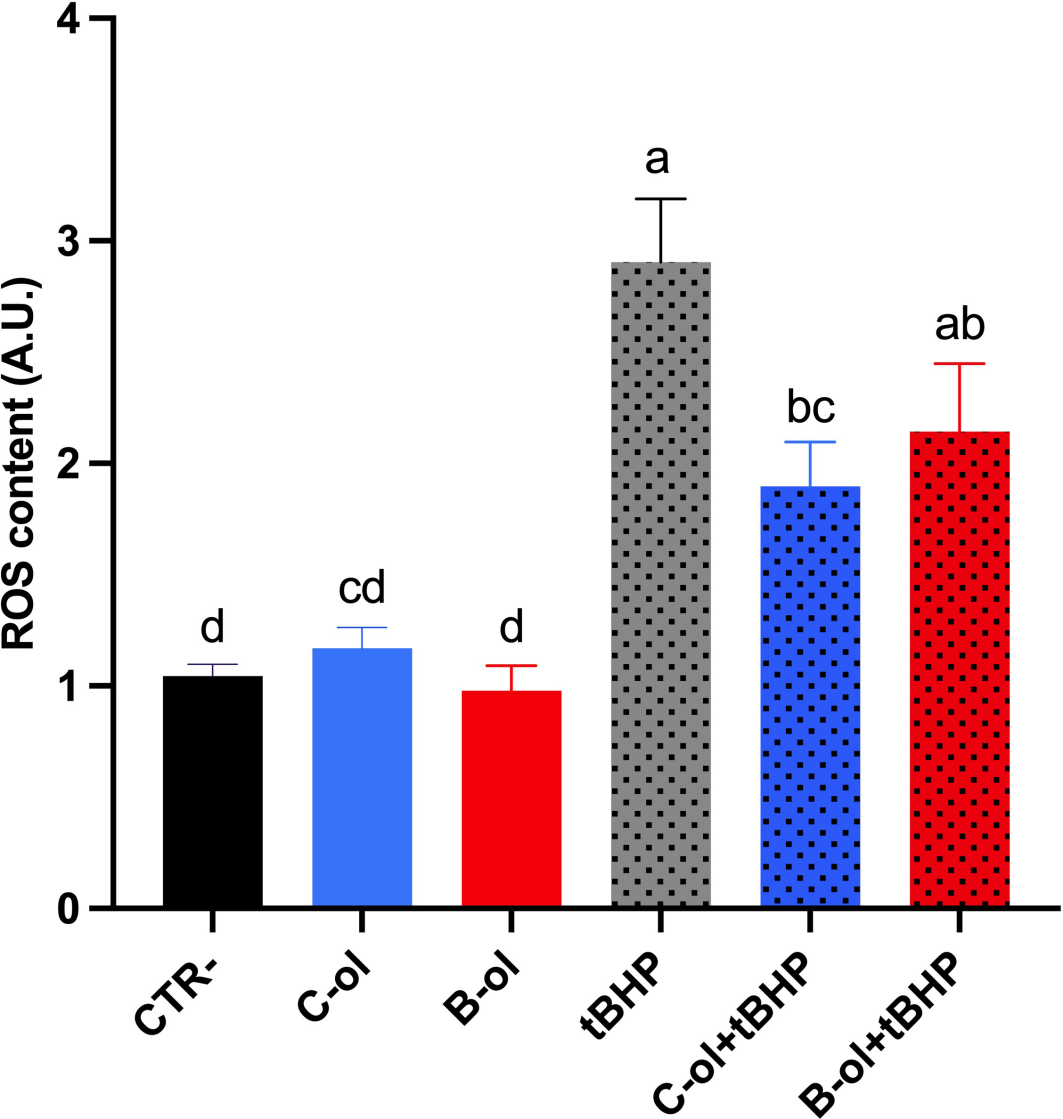
ROS content measured using dihydrorhodamine-123 fluorescence in MCD4 cells treated for 24 h at concentration of 1µg/mL of total phenol content of olive leaf extract samples. Data are shown as mean ± SEMs and analysed by one-way ANOVA followed by Tukey’s multiple comparison test. Data are expressed as arbitrary units (A.U.) of fluorescence intensity normalized to control cells. Different letters mean a significant difference (*p ≤* 0.05) between samples. Abbreviations: C: Coratina olive leaf extract, B: Bambina olive leaf extract, tBHP, tert-butyl hydroperoxide.

To evaluate the antioxidant activity of the olive leaf extracts, MCD4 cells were treated as described above, and intracellular ROS content was measured. As shown in **Figure 6**, treatment with Bambina and Coratina olive leaf extracts did not increase basal ROS levels, suggesting that neither extract has a pro-oxidant effect under basal conditions. However, when compared with cells treated with tBHP, incubation with olive leaf extracts reduced the intracellular ROS levels induced by tBHP. Specifically, C-ol significantly reduced ROS content compared to tBHP, indicating a clear antioxidant effect. In contrast, B-ol showed a tendency towards reduction, although the difference did not reach statistical significance.

## 4 Discussion

Virgin olive oil (VOO) is obtained exclusively by mechanical extraction from olive fruits, preserving a complex matrix of lipid and minor bioactive compounds that contribute to its nutritional, functional, and sensory properties (Jimenez-Lopez et al., 2020). The quality of VOO is largely determined by its fatty acid profile, primarily by oleic acid content, and by the presence of antioxidant minor compounds such as phenols, tocopherols, carotenoids, and volatile molecules. These compounds play a key role in oxidative stability, health-related properties, and characteristic sensory attributes, including bitterness, pungency, and fruity notes. However, the relative abundance and qualitative composition of these compounds are strongly influenced by the specific cultivar and processing conditions. In this context, *cv* Coratina is widely recognized for its high phenolic content and pronounced sensory intensity (Campestre et al., 2017; Recinella et al., 2022), whereas autochthonous cultivars such as *cv* Bambina remain comparatively under-characterized, despite their potential to express valuable nutraceutical and sensory properties when processed under optimized technological conditions. Therefore, the application of a nutraceutical milling strategy represents a key approach to enhance the antioxidant composition of VOO, aiming to achieve chemical and biological properties comparable to those of recognized high-quality cultivars. In this perspective, technological efforts have been directed toward improving both the extraction efficiency of biophenols and their post-extraction preservation, with particular focus on process variables affecting biophenol content during malaxation, including time, temperature, atmosphere composition, and water addition. High temperatures may negatively affect phenolic content by promoting oxidative degradation, whether enzymatically driven or via chemical pathways. Nevertheless, findings reported in the literature are not always consistent with this view. Some studies have shown that, at the industrial scale, elevated temperatures can enhance the partitioning of hydrophilic phenols into the oil phase (Boselli et al., 2009; Guerrini et al., 2019). In contrast, other studies have demonstrated that lower malaxation temperatures may favour higher oil phenolic content (Marx et al., 2021).

In the present study, *cv* Coratina, a widely cultivated variety in the Apulia region, was used as a reference cultivar to comparatively evaluate the nutraceutical potential of *cv* Bambina. In addition, the impact of an advanced oil extraction method, based on reduced malaxation temperatures and shortened kneading time, was assessed with respect to the retention of bioactive compounds and the overall nutritional quality of the resulting oils. As shown in **Table 1**, the modified extraction process enhanced both total tocopherols and phenolic compounds in the oils of both cultivars, irrespective to cultivar identity. Indeed, regardless of the absolute differences in concentration, attributable to cultivar-specific characteristics (Miho et al., 2018; Squeo et al., 2025), the nutraceutical process significantly increased biophenols and tocopherols in both cases. Consistently with Marx et al. (2021), both free and esterified forms of biophenols were affected. Notably, this positive trend was also confirmed when the kneading time was drastically reduced to as little as 5 minutes. These results suggest that the observed enhancement in antioxidant bioactives in both Coratina and Bambina VOOs is attributable to the combined effect of reduced malaxation time and temperature. Specifically, shorter kneading time limits the window available for endogenous oxidoreductases to enzymatically degrade these compounds, while lower temperatures establish sub-optimal working conditions for the same enzymes, namely peroxidase and polyphenol oxidase, whose optimal activity temperatures have been reported to be considerably higher (Clodoveo et al., 2014). Finally, the increase observed in phenolic alcohols (hydroxytyrosol and tyrosol) under nutraceutical extraction conditions should be interpreted as evidence of effective protection against oxidation rather than enhanced hydrolysis of more complex secoiridoids derivatives into simpler forms. Hydroxytryrosol is recognized as one of the most potent antioxidants in VOOs (Servili et al., 2009).

It is noteworthy that the extraction trials were already carried out at industrial scale, underscoring the immediate scalability and applicability of the innovative process described here. Temperature management during VOO extraction has become a key feature of high quality production since several years, and controlled-temperature equipment such as crushers, heat exchangers, etc., has been extensively investigated in this context (Kalogianni et al., 2019; Nucciarelli et al., 2022). Such solutions are already commercially available and ready to be implemented in real industrial scenarios.

The phenolic profiles and tocopherol contents observed in both *cv* Bambina and *cv* Coratina VOOs acquire additional significance when placed in the broader context of Mediterranean olive diversity. Widely cultivated Spanish varieties such as *cv* Picual and *cv* Arbequina are well known for their agronomic performance but typically yield oils with moderate phenolic levels compared to Southern Italian high-polyphenol varieties (Cert et al., 2000; Miho et al., 2018). In contrast, some Portuguese, Greek and Turkish cultivars have been reported to accumulate phenolic contents broadly comparable to those of *cv* Coratina under standard extraction conditions (Vinha et al., 2005; Köseoğlu et al., 2016; Georgiadou et al., 2019). Notably, nutraceutical milling elevated the total biophenol content of *cv* Bambina oil to levels approaching those commonly reported for *cv* Koroneiki, positioning this autochthonous Apulian cultivar competitively within the Mediterranean landscape of high-value VOO production. Tocopherol concentrations under nutraceutical conditions were also consistent with or exceeding the upper range documented for major Mediterranean varieties (Georgiadou et al., 2019), further supporting the nutraceutical relevance of the optimized extraction strategy.

To further explore the antioxidant potential of *cv* Bambina oil considering the promising results obtained with nutraceutical milling, a combination of molecular and antioxidant activity assays was performed. The transcriptional profiles of genes involved in vitamin E biosynthesis were investigated and compared between *cv* Bambina and *cv* Coratina drupes. At early-intermediate fruit ripening, upstream genes (*OeVTE5*, *OeGGR*, *OeHPPD*) exhibited differential regulation relative to downstream genes (*OeVTE2*, *OeVTE3*, *OeVTE1*, *OeVTE4*), particularly in *cv* Bambina. The pronounced upregulation of *OeGGR* and *OeVTE5* is consistent with prior reports linking temporal accumulation of vitamin E to cultivar-dependent activation of upstream pathway genes (Georgiadou et al., 2015, Georgiadou et al., 2016 and Georgiadou et al., 2019; Narvàez et al., 2025). This likely reflects increased phytol recycling from chlorophyll catabolism during fruit maturation, thereby fuelling phytyl-PP synthesis and subsequently enriching tocochromanol accumulation (Muñoz and Munné-Bosch, 2019). Transcript levels of the downstream genes *OeVTE2* and *OeVTE3* were comparable to those reported in other olive cultivars at equivalent ripening stages (Navàez et al., 2025) and in other plant species under cold stress conditions (Rey et al., 2021; Zeng et al., 2023), supporting their conserved functional role during fruit development across species.

Among lipoxygenases, which are key enzymes in VOO aroma formation, the expression profiles of two late-ripening genes, *OeLOX* and *Oe2LOX2*, were investigated. *OeLOX* exhibited markedly higher transcript levels in *cv* Bambina than in *cv* Coratina, consistent with its primary role in enhancing aromatic complexity and sweet sensory notes characteristic of Bambina oil, in agreement with observations reported in other olive cultivars (Palmieri-Thiers et al., 2009; Camarero et al., 2023). Conversely, *Oe2LOX2*, which encodes a chloroplast-localized, jasmonate-related isoform identified by Padilla et al. (2009) as the main contributor to VOO volatile biosynthesis in Picual and Arbequina cultivars, showed lower expression level than *OeLOX* in both cultivars examined here. *OePOD* and *OePOD42L*, encoding two peroxidase isoforms, were more highly expressed in *cv* Bambina than in *cv* Coratina, although these differences did not reach statistical significance. Collectively, this pattern of gene expression highlights intrinsic varietal characteristic of *cv* Bambina that may differentially modulate both aroma-related enzymatic pathways and antioxidant capacity. These findings were corroborated by gene expression analysis in pastes obtained from both milling procedures. Results under standard condition mirrored the profiles observed in drupes, with *cv* Bambina consistently showing the highest expression levels. Interestingly, nutraceutical milling appeared to up-regulate *OeLOX* transcription while negatively affected *OePOD* transcription.

Following gene expression analysis, the activity of the corresponding enzymes was evaluated in olive pastes of both cultivars obtained through both extraction methods. The activity of endogenous oxidative enzymes in olive pastes is a key determinant of both the antioxidant profile and the sensory quality of VOO. Peroxidase (POD) and polyphenol oxidase (PPO) are primarily involved in the oxidation of phenolic compounds, reducing oil stability and nutraceutical value, whereas lipoxygenase (LOX) plays a dual role: it is associated with lipid oxidation as well as with the biosynthesis of C6 volatile compounds responsible for the positive green and fruity sensory notes characteristic of high-quality VOO. The results demonstrate that nutraceutical milling markedly affected the enzymatic profile of olive pastes, with both process- and cultivar-dependent responses. A significant reduction in POD activity was observed in pastes of both cultivars processed by the nutraceutical method, consistent with the low-temperature conditions and shortened malaxation time applied, which likely established sub-optimal conditions for POD activity. These findings further support the notion that stringent control of technological parameters is crucial to limiting oxidative degradation and preserving antioxidant molecules during oil extraction. In contrast, PPO activity showed no significant variations between cultivars or extraction methods, possibly indicating a limited contribution of PPO to phenolic oxidation at the considered ripening stage, or a lower sensitivity of this enzyme to the applied processing conditions.

A distinct trend was observed for LOX activity, which was selectively enhanced in *cv* Bambina under nutraceutical milling conditions while remaining unchanged in *cv* Coratina. This cultivar-specific response is particularly noteworthy, as it mirrors the transcriptional upregulation of *OeLOX* detected in both Bambina drupes and pastes. The increased LOX activity in *cv* Bambina suggests a greater intrinsic capacity to activate the lipoxygenase pathway under mild processing conditions, potentially promoting the biosynthesis of C6 volatile compounds associated with the characteristic aromatic complexity and desirable sensory attributes of the resulting oil.

Overall, the combined analysis of gene expression and enzymatic activities indicates that *cv* Bambina exhibits a distinctive oxidative-antioxidant balance during processing. While this cultivar appears intrinsically more responsive at the enzymatic level, the nutraceutical milling strategy effectively curtails POD-mediated oxidative reactions while preserving or enhancing LOX-related aroma-forming pathways. This enzymatic modulation, together with the enhanced retention of phenolic compounds and tocopherols, helps explaining the improved nutraceutical and sensory quality of *cv* Bambina oil obtained through the modified extraction process.

To assess whether these biochemical and technological differences translate into a measurable biological effect, the antioxidant potential of digested oils was subsequently evaluated using an *in vitro* cellular model. Renal collecting duct cells (MCD4) were selected as a sensitive system to assess intracellular oxidative stress modulation upon exposure to digested olive oil samples and olive leaf extracts. Intracellular ROS levels were detected in cell lysates using a fluorescent probe to improve quantitative accuracy and reproducibility. Notably, this approach minimizes potential interfering factors associated with live-cell measurements, such as differences in cell dye loading, cell density, and probe compartmentalization. Under basal conditions, none of the digested oil samples altered intracellular ROS levels, confirming the absence of pro-oxidant effects and the cytocompatibility of both *cv* Bambina and *cv* Coratina digested oils, independently of the extraction method applied.

When oxidative stress was induced by tert-butyl hydroperoxide (tBHP), digested oils from both cultivars significantly reduced intracellular ROS accumulation, demonstrating a clear antioxidant and cytoprotective effect. No substantial differences were detected between oils obtained by standard and nutraceutical milling, nor between Bambina and Coratina cultivars. Despite the higher phenolic and tocopherol content measured in nutraceutical oils, the overall antioxidant capacity at the cellular level converged toward a comparable protective threshold under the experimental conditions employed. This outcome likely reflects the complexity of intracellular antioxidant mechanisms, in which bioavailability, cellular uptake, and synergistic interactions among biocompounds may attenuate the quantitative differences observed at the chemical level (Servili et al., 2013).

A similar trend was observed in experiments conducted with olive leaf extracts. Neither *cv* Bambina nor *cv* Coratina extracts elevated basal ROS levels, confirming the absence of pro-oxidant activity. Under oxidative challenge, the *cv* Coratina olive leaf extract significantly reduced tBHP-induced ROS production, whereas the *cv* Bambina extract showed a comparable but statistically non-significant trend toward ROS reduction. This difference may be attributable to the markedly higher total phenolic content of Coratina leaves, which likely confers a stronger antioxidant capacity under acute oxidative stress conditions (Kontogianni and Gerothanassis, 2012; Caponio et al., 2025a). Although phenolic compounds, particularly oleuropein, are considered the primary contributors to antioxidant activity, olive leaf extracts also contain other bioactive molecules, including triterpenes, organic acids, and minor constituents, which may contribute individually or synergistically to the observed biological effects (Selim et al., 2022).

Overall, these cellular data demonstrate that both digested oils and olive leaf extracts from *cv* Bambina and *cv* Coratina exert a protective antioxidant effect against induced oxidative stress, notwithstanding the cultivar- and process-dependent differences observed at the biochemical and molecular levels. Importantly, the comparable ROS modulation across treatments supports the conclusion that *cv* Bambina-derived products achieve a biological antioxidant efficacy equivalent to that of the well-established *cv* Coratina. Future studies should reinforce the robustness of these findings by applying the nutraceutical milling process to other autochthonous olive genotypes and through multi-seasonal trials, given that climatic conditions are known to substantially influence the phenolic and tocopherol composition of VOO. In addition, the antioxidant bioactivity of the resulting oils should be assessed across a broader range of *in vitro* cellular models, and dose-response bioavailability experiments should be conducted to better define the translational relevance of the observed effects.

## 5 Conclusions

This research highlights the qualitative and functional properties of the autochthonous *cv* Bambina, demonstrating that its oil achieves a nutraceutical and biological antioxidant potential comparable to that of the renowned *cv* Coratina. The nutraceutical milling strategy significantly enhances the retention of phenolic compounds and tocopherols while favourably modulating endogenous enzymatic activities, thereby improving both the oxidative stability and the overall quality of the oil. Importantly, *in vitro* cellular assays confirmed that digested oils and olive leaf extracts from *cv* Bambina effectively counteract tBHP-induced oxidative stress. Collectively, these results establish *cv* Bambina as a valuable genetic and nutraceutical resource, worthy of valorisation within sustainable olive oil production system and of inclusion in future breeding as well as in biodiversity conservation programs.

## Data Availability

The raw data supporting the conclusions of this article will be made available by the authors, without undue reservation.
