# Supplementary material for: Biological evaluation and nutraceutical potential of Bambina, a resilient Apulian olive cultivar, through an advanced milling process
Source: Front Plant Sci. 2026 May 1;17:1815003. doi: 10.3389/fpls.2026.1815003 (PMC13176157; doi:10.3389/fpls.2026.1815003)
Supplement: Supplementary file 1 [file SupplementaryFile1.docx]

Supplementary Material

**Supplementary references**

Alagna, F., Mariotti, R., Panara, F., Caporali, S., Urbani, S., Veneziani, G., et al. (2012). Olive phenolic compounds: metabolic and transcriptional profiling during fruit development. BMC Plant Biol. 12:162. doi: 10.1186/1471-2229-12-162.

Dastkar, E., Soleimani, A., Jafary, H., Karimi, G., Gharechahi, J., Salekdeh, G. H., et al. (2020). Differential expression of genes in olive leaves and buds of ON- versus OFF-crop trees. Sci. Rep. 10:15762. doi: 10.1038/s41598-020-72895-7.

Georgiadou, E. C., Ntourou, T., Goulas, V., Manganaris, G. A., Kalaitzis, P., and Fotopoulos, V. (2015). Temporal analysis reveals a key role for VTE5 in vitamin E biosynthesis in olive fruit during on-tree development. Front. Plant Sci. 6:871. doi: 10.3389/fpls.2015.00871.

Muzzalupo, I. (2012). "Olive germplasm - Italian catalogue of olive varieties," in Olive Germplasm - Italian Catalogue of Olive Varieties, ed. I. Muzzalupo (Rijeka: InTech). doi: 10.5772/51719.

Padilla, M. N., Hernández, M. L., Sanz, C., and Martínez-Rivas, J. M. (2009). Functional characterization of two 13-lipoxygenase genes from olive fruit in relation to the biosynthesis of volatile compounds of virgin olive oil. J. Agric. Food Chem. 57, 9097–9107. doi: 10.1021/jf901777j.

Sánchez, R., Arroyo, L., Luaces, P., Sanz, C., and Pérez, A. G. (2023). Olive polyphenol oxidase gene family. Int. J. Mol. Sci. 24:3233. doi: 10.3390/ijms24043233.
