# Supplementary material for: Biological evaluation and nutraceutical potential of Bambina, a resilient Apulian olive cultivar, through an advanced milling process
Source: Front Plant Sci. 2026 May 1;17:1815003. doi: 10.3389/fpls.2026.1815003 (PMC13176157; doi:10.3389/fpls.2026.1815003)
Supplement: Supplementary file 2 [file Table1.docx]

Supplementary Material

**Table S1.** List of genes, accession numbers, and primer pairs used for gene expression analysis in this study.

| **Gene** | **Accession n.** | **Forward sequence** | **Reverse sequence** | **Reference** |
| --- | --- | --- | --- | --- |
| *OeGGR* | DQ424963 | CCAAGGGAGGCATTTGTAGA | TGGATTCACAGCCAATTTCA | Georgiadou et al., 2015 |
| *OeHPPD* | E8NTSAO02CY14E | CCCTTCATTGTGCTCCAAGT | CCACTGGATTCCATGAATTTG | Georgiadou et al., 2015 |
| *OeHGGT* | E8NTSAO04IZCRI | AATATCCTCCCGATGCCAAT | ACGGCCCCACACAGTAATAG | Georgiadou et al., 2015 |
| *OeVTE1* | E8NTSAO03HE119 | TCAACACATGGAAAGGCAAG | CATTTATGGTGCTGCAGGTG | Georgiadou et al., 2015 |
| *OeVTE2* | E8NTSAO01BSBQ9 | TTGCATTGAGTCCAAGATGC | GAAATTATGCTCAATGCTGTTCC | Georgiadou et al., 2015 |
| *OeVTE3* | E8NTSAO01BS0YR | CCCACGATACCATTTTGGAC | GCTTGCGTAATTGGTCCTGT | Georgiadou et al., 2015 |
| *OeVTE4* | E8NTSAO03GW1S2 | TTCCGGCATGGTGTTCTACT | GGCCAGTGCTCCTTTTATTG | Georgiadou et al., 2015 |
| *OeVTE5* | E8NTSAO03HA0AH | CTGCTTTGTCGTTGGAGAGA | TCAGTAGCCAAGGAAAGACCA | Georgiadou et al., 2015 |
| *OePOD* | XM_022996091.1 | CTACAGACGTACAAAGCGCC | CCCATCTTCAGCCCGTCTAT | Authors of this manuscript |
| *OePOD42like* | XM_023022187.1 | CCGCTCAAGAACAACATT | AACTGGAAGAAGAGATGGT | Dastkar et al., 2020 |
| *OePPO1* | MW038828 | AGTGTACTGCTGCCGGAAGTTTG | TGTTGATTTGGAACCAACCACCT | Sanchez et al., 2023 |
| *OePPO3* | OL870608 | CGATGAAGAAAGTACGCCGGA | ACCAAAGTCACCAAAACATGCT | Sanchez et al., 2023 |
| *OeLOX* | EU678670 | TCCCATTGCCTCAGGTTATCA | TCTCTCGCGAATTCTTCATCTG | Muzzalupo et al., 2012 |
| *Oe2LOX2* | EU513353 | TCGCTGGGAAAGTGAAAGAG | TCAAATGGAAACGCTGTTAGG | Padilla et al., 2009 |
| *OeEF1a* | AM946404 | ACCACTGGTGGTTTTGAAGC | GAAACCAGAGATGGGGACAA | Alagna et al., 2012 |
