# Supplementary material for: Biological evaluation and nutraceutical potential of Bambina, a resilient Apulian olive cultivar, through an advanced milling process
Source: Front Plant Sci. 2026 May 1;17:1815003. doi: 10.3389/fpls.2026.1815003 (PMC13176157; doi:10.3389/fpls.2026.1815003)
Supplement: Supplementary file 3 [file Table2.docx]

Supplementary Material

**Table S2.** Total phenol content (TPC) of olive leaf extracts.

|  | **C-ol** | **B-ol** |
| --- | --- | --- |
| TPC (µg GA mL^-1^) | 2242.29 ± 6.70^a^ | 661.10 ± 2.00^b^ |

Data are expressed as mean ± SD, with different superscript letters indicating a significant difference (*p* < 0.05; two-tailed Student’s *t*-test). Abbreviations: C-ol, Coratina olive leaf extract; B-ol, Bambina olive leaf extract, GA, gallic acid, TPC, total phenol content.
